# An Increased Total Resected Lymph Node Count Benefits Survival following Pancreas Invasive Intraductal Papillary Mucinous Neoplasms Resection: An Analysis Using the Surveillance, Epidemiology, and End Result Registry Database

**DOI:** 10.1371/journal.pone.0107962

**Published:** 2014-09-29

**Authors:** Wenming Wu, Xiafei Hong, Rui Tian, Lei You, Menghua Dai, Quan Liao, Taiping Zhang, Yupei Zhao

**Affiliations:** Department of General Surgery, Peking Union Medical College Hospital, Chinese Academy of Medical Sciences, Beijing, China; and Peking Union Medical College, Beijing, China; The University of Hong Kong, China

## Abstract

**Background:**

The therapeutic effect of lymph node dissection for pancreas invasive intraductal papillary mucinous neoplasms (IPMN) remains unclear. The study investigated whether cancer-specific survival (CSS) and overall survival (OS) rates among invasive IPMN patients improve when more lymph nodes are harvested during surgery.

**Study Design:**

The study cohort was retrieved from the Surveillance, Epidemiology, and End Results (SEER) database. The lymph node count was categorized into quartiles. The relationship between lymph node count and survival was analyzed using Kaplan–Meier curves and a Cox proportional-hazards model. The stage migration was assessed by Chi-square tests. Propensity score matching (PSM) was used to minimize confounding variables between groups.

**Results:**

In total, 1,080 patients with resected invasive IPMNs from 1992 to 2011 were included. Univariate and multivariate Cox models indicated that an increased lymph node count independently improves survival. The Kaplan-Meier and log-rank tests identified 16 nodes as an optimal cut-off value that yielded a significant survival benefit for all invasive IPMN patients. The stage migration effect existed in this cohort. After PSM, the 5-year CSS increased from 36% to 47%, and the median survival rate increased from 30 months to 40 months by increasing the lymph node count to over 16, alone. The 5-year OS rate also provided additional support for this result.

**Conclusion:**

Increased lymph node counts were associated with improved survival in invasive IPMN patients. One cut-off value of lymph node count was 16 for this improvement.

## Introduction

Intraductal papillary mucinous neoplasms (IPMN) have been increasingly recognized as an intraductal mucin-producing pancreatic neoplasms with tall, columnar mucin-containing epithelium and a lack of ovarian stroma, according to the World Health Organization definition [1], [2]. The estimated IPMN prevalence is around 26 per 100,000 people, and the prevalence of this disease is roughly 99 per 100,000 people in the population over 60 years old [3],[4]. Approximately 5% of all pancreatic cancers are invasive IPMNs from an analysis from the Surveillance, Epidemiology, and End Result (SEER) database [5].

IPMN can be classified into adenoma, borderline dysplasia, carcinoma in situ and invasive lesions based on the cellular atypia degree [6]. The three major IPMN types are the main duct-type, branch duct-type, and the mixed-type, based on their relationships with the main pancreatic duct [7]. The common main duct-type IPMN features include main pancreatic duct obstruction and dilatation [8]. Approximately 40%–80% of main duct-type IPMNs are invasive lesions [9]–[12]. Branch duct-type IPMNs reside in the branches of the pancreatic duct. Approximately 10%–30% of branch duct-type IPMNs are invasive lesions and are much less frequent than main duct-type IPMNs [9]–[14]. The International Consensus Guidelines recommended resection for all main duct-type IPMNs and certain branch duct-type IPMNs with suspicious malignant features [15], [16]. The prognosis is poorer for invasive IPMNs compared with their non-invasive counterparts. The five-year survival rate was estimated to be nearly 31%–41% for invasive IPMNs and 77%–94.5% for non-invasive IPMNs [17]–[19]. The recurrence rate is also higher for invasive IPMNs (30%–65%) compared with non-invasive IPMNs (6–14%) [17], [19]–[21]. Distance metastases are common for invasive IPMN recurrences and usually involve the liver, peritoneum, and other abdominal organs. Local recurrences in the remnant pancreas are seen in some patients who had a partial pancreatectomy as an initial treatment.

The lymph node status has long been recognized as the prognostic indicator for invasive IPMNs. Survival is even worse for those with positive nodes compared with negative ones [9], [22]. Moreover, a higher lymph node ratio is associated with a poorer prognosis [23]. Lymph node dissections during surgery could be a potential therapeutic strategy for lymphatic invasive IPMN metastases. The total examined lymph node count can be a surrogate indicator for the lymph node dissection extent. Thus, in the present study, we aim to explore the association between the number of lymph nodes harvested and the patient survival rate following an invasive IPMN using the SEER database.

## Methods

### SEER Database

The SEER program is an authoritative American cancer information database. As provided by the National Cancer Institute, the SEER program collects and publishes cancer data from 18 population-based cancer registries among 14 states across the United States. It gathers data on cancer incidence, patient demographics, and mortality, which include the primary sites, histological diagnoses, grading, morphology, pathological staging, first treatment course, and vital statuses. The SEER database is collected and released annually, reflecting the latest updated information. We obtained data from the SEER 18 Registry, and the including criteria follows the current classification scheme. SEER 18 was submitted in April 2014, with the broadest coverage (28%) of the population ever released [24].

### Case Selection

The study utilized SEER*Stat version 8.1.5 to create the case list of interest, stratify the data and to reveal any correlations. We initially identified our patient cohort by querying the “SEER Site Recode” with the term “pancreas” as the primary disease site. Then, we identified invasive IPMNs using the variable “Histologic Type ICD-O-3” (International Classification of Disease for Oncology, 3rd Edition) through codes defined by the ICD-O-3, which included 8050, 8260, 8450, 8453, 8471, 8480, 8481, and 8503. Our initial population included 8,228 patients. Among the population, we included patients with year-of-diagnosis between 1992 and 2011 and underwent IPMN-directed surgery. Those that did not undergo surgery or underwent non-IPMN-directed surgery were excluded.

For cancer staging, we adopted the AJCC (American Joint Committee on Cancer) 6th edition definition as the criterion, which has routinely been reported in the SEER database since 2004. We did not adopt the AJCC 7^th^ edition definition because it was only routinely reported for the cases from 2010 to present. As for the patients diagnosed between 1992 and 2003, there was no direct AJCC TNM system from the database. Instead, we analyzed the extended IPMN information fields, such as tumor morphology, tumor size, disease extent, and nodal status, to assign a suitable AJCC (6th edition) stage definition to the included IPMN cases. This study was approved by the Ethical Committee of Peking Union Medical College Hospital. Written informed consents were not obtained from participants for their clinical records to be used in this study by our hospital. We confirmed that patient records and information were anonymized and de-identified prior to analysis.

### Materials and data

The baseline information factors for the statistical model were derived from two main sources. These factors included patient demographic characteristics, including age, sex, race, marital status, diagnosis year, as well as the tumor and surgical characteristics, including the tumor location (pancreatic head or other), T stage (T1- T4, or unknown), N stage/node status (N0, N1, or unknown), dissected lymph node number, positive node number, M stage (M0, M1, or unknown), AJCC staging (stage 1–4, or unknown), histological grade (grade I-IV, or unknown), surgical procedures (pancreatoduodenectomy, total pancreatectomy, partial, or local resection), and information regarding the receipt of radiotherapy. Patients with a live vital status or lost to follow-up were right-censored for the overall survival (OS) analysis, and those whose cause of death was not attributable to pancreatic etiology were right-censored for the cancer-specific survival (CSS) analysis.

### Statistical analysis, survival analysis and stage migration detection

The primary endpoint was the 5-year CSS rate. In the first model, the lymph node count was analyzed as a continuous variable. Before univariate analysis, we tested if the correlations among any variables were strong, which was defined as collinearity [25]. We calculated variance inflation factors (VIF) as an indicator for collinearity (VIF>4 as significant collinearity). In detail, the survival month was used as the dependent factor and all variables mentioned in the previous ‘Material and Data’ section were used as predictors for the dependent factor. Then any variables with VIF>4 were removed from further analysis. A univariate analysis was performed and variables with P value less than 0.2 were enrolled into multivariate Cox proportional hazards regression model to obtain an adjusted hazard ratio (HR) and 95% confidence intervals (CI). To explore the optimal lymph node count cut-off, we stratified the entire cohort into four subgroups that had an approximately equal patient number. The lymph node count intervals in the four groups were 1–5, 6–10, 11–16, and>16. The Kaplan-Meier method was utilized to generate survival curves for the four lymph node count intervals and log-rank tests were utilized to calculate P values for group comparisons. Based on this, the optimal cut-off value should separate the four intervals into two groups. The two intervals in different groups should be statistically significant by the log-rank test, while the two intervals in the same groups should not. In the second model, the lymph node count was analyzed as a dichotomous variable based on the optimal cut-off value. Univariate and multivariate analyses were conducted in the same manner as the first model. To explore the relationship between lymph node count and survival in different T, N stages and histological grades, subgroup analyses were conducted for each T, N stage and histological grade. To estimate any potential stage migration effect, we used a cross table to assess the percentage of T stage, N stage and histological grade for each four lymph node count interval. Chi-square analyses were used to determine any statistically significant differences between intervals. The entire statistical analysis was performed with the SPSS software, version 20.0 (SPSS, Chicago, IL, USA). All reported P values were two-sided, and the statistical significance level was set at P under 0.05.

### Propensity Score Matching (PSM)

The SEER database is a nonrandomized cohort. Therefore, we used propensity score matching to adjust the lymph node count for a set of pre-test covariates. The aim of this approach was to balance the covariates between the patients with greater lymph node counts and those with fewer ones, therefore, mimicking nonrandomized study with its randomized counterpart [26], [27]. A set of covariates was predefined as independent variables. The provision of a lymph node dissection with a total count of>16 was set as the dependent variable. The propensity score was estimated using a multivariate logistic regression model. Kaplan-Meier survival curves were generated after matching for both CSS and OS. The PSM was conducted using the “psmatching” program, which employed an SPSS interface to run the analysis in R [28].

## Results

### Baseline Characteristics of the Cohort

Among the initial population of 8,228 patients, a total of 1,080 patients were enrolled in the study cohort after application of the inclusion and exclusion criteria. The average age of the cohort was 65.2 years with a standard deviation of 12.1 years. Among the cohort, 45.7% were female, 84.0% were white, and 66.9% were married (Table 1). The diagnosis years (1992–2011) were classified every fifth year, and as time passed by, the five-year diagnosis count grew larger. For the latest five-year interval, 2007 to 2011, the IPMN patient sum accounted for 37.6% of the entire cohort. 65.8% of the tumors located at the pancreas head. For the T stages, stage T3 constituted more than half of the tumors (56.4%), followed by T2 (21.5%), T1 (11.9%), and T4, which contributed the smallest proportion (3.7%). For the N stages, which stand for the lymph node status, there were more patients with N0 stage were involved (54.4%) than the N1 stage. For the M stages, which represent the distant metastasis condition, the patients with M0 stage constituted the majority (93.1%). As for the AJCC staging system, there was a predominance of stage II tumors (61.1%). Regarding the histological grading, there were more Grade II than Grade I and III tumors. As far as clinical treatments, pancreatoduodenectomy preponderated (68.0%) as a surgical therapy; Meanwhile, 31.8% patients received radiotherapy. In our study, 34.4% of the patients were still alive at the time of analysis, while the remaining 65.6% were dead. This was mainly attributed to the pancreatic etiology fatality rate (53.9%). The number of lymph nodes dissected varied from 1 to 70, with an average of 12 and a median of 10. Thereof, we assigned the cohort into quartiles to yield four groups with nearly the same patient number. The lymph node number and relative percentage of each quartile were 1–5 (25.9%), 6–10 (26.1%), 11–16 (23.8%), and over 16 (24.2%). The number of positive nodes varied from 0 to 34, but averaged only 1.4 nodes.

**Table 1:** Patient, Tumor, and Treatment Characteristics in the Invasive IPMN Patients: Surveillance, Epidemiology, and End Results, 1992 to 2011.

Variable	Patients No.	Percentage
**Age, y**		
<65	454	42.0
≥65	626	58.0
**Sex**		
Women	494	45.7
Men	586	54.3
**Race**		
White	907	84.0
Black	78	7.2
Other (American Indian/AK Native, Asian/Pacific Islander)	91	8.4
Unknown	4	0.4
**Marital status**		
Married	723	66.9
Other	357	33.1
**Diagnosis years intervals**		
1992–1996	109	10.1
1997–2001	209	19.4
2002–2006	356	33.0
2007–2011	406	37.6
**Tumor location**		
Pancreatic Head	711	65.8
Other	369	34.2
**T stage**		
T1	129	11.9
T2	232	21.5
T3	609	56.4
T4	40	3.7
Unknown	70	6.5
**N stage**		
N0	588	54.4
N1	483	44.7
Unknown	9	0.8
**M stage**		0.0
M0	1005	93.1
M1	67	6.2
Unknown	8	0.7
**AJCC stage**		
I	274	25.4
II	660	61.1
III	28	2.6
IV	75	6.9
Unknown	43	4.0
**Histological Grade**		
Well differentiated; Grade I	213	19.7
Moderately differentiated; Grade II	421	39.0
Poorly differentiated; Grade III	196	18.1
Undifferentiated; Grade IV	11	1.0
Unknown	239	22.1
**Surgery Type**		
Pancreatoduodenectomy	734	68.0
Total pancreatectomy	137	12.7
Partial or Local pancreatectomy	180	16.7
Other	29	2.7
**Radiation therapy**		
Received	343	31.8
Not Received	718	66.5
Unknown	19	1.8
**Cause of death**		
Alive	372	34.4
Death	708	65.6
Pancreas	582	53.9
Other cause of death	126	11.7
**Lymph Nodes Examined**		
1∼5	280	25.9
6∼10	282	26.1
11∼16	257	23.8
>16	261	24.2

### The Correlation between the Dissected Lymph Node Number and Survival Time

We used univariate survival and multivariate Cox survival analyses to identify the correlation between the CSS and the variables mentioned above. The continuous variables included age, diagnosis year, dissected lymph node number, and the positive lymph node number. The categorical variables, included sex, race, marital status, tumor location, T stage, N stage, M stage, AJCC stage, pathological grade, surgical procedure, and information regarding the receipt of radiotherapy.

Before univariate analysis, we calculated VIF using survival time as the dependent factor, and all variables mentioned above were used as predictors for the dependent variable. After the initial analysis, only AJCC stage had a VIF over 4 (VIF  = 6.27), and all other variables had VIFs less than 4. Therefore, AJCC stage was strongly correlated with other variables and was excluded from further analysis. After the exclusion, we re-conducted the VIF calculation for the other variables, and all variables had VIFs close to 1, indicating no strong correlation between the remaining variables (Table S1).

Through the univariate analysis, the variables with P value greater than 0.2 were sex, marital status, tumor location, and surgical procedures; therefore, these variables were no longer preserved for the following multivariate analysis. The rest of the variables proceeded into the multivariate Cox survival analysis. We discovered several independent risk factors, including advanced age, high T stage, nodal metastasis, distant metastasis, advanced pathological grade, and increased in positive lymph nodes. Additionally, independent protective factors, including race (American Indian/AK Native, Asian/Pacific Islander), late diagnosis year, and an increased dissected lymph node number, were also identified. It was notable that these variables revealed that the dissected lymph node number was an independent protective factor, whereby the HR with each additionally removed lymph node was 0.966 (95% CI: 0.952–0.979, p<0.001) (Table 2).

**Table 2:** Univariate and Multivariate Cox Proportional Hazard Regression Analyses for Cancer-Specific Survival in Invasive IPMN Patients: Surveillance, Epidemiology, and End Results, 1992 to 2011.

	Univariate analysis		Multivariate analysis	
	HR (95% CI)	p value	HR (95% CI)	p value
**Age, y (continuous variable)**	1.017 (1.010–1.024)	**<0.001**	1.017 (1.008–1.026)	**<0.001**
**Sex**		0.581		
Women	1.0 (referent)			
Men	0.955 (0.812–1.124)			
**Race**		**0.004**		**0.019**
White	1.0 (referent)		1.0 (referent)	
Black	0.861 (0.616–1.202)	0.378	0.947 (0.648–1.383)	0.777
Other (American Indian/AK Native, Asian/Pacific Islander)	0.563 (0.400–0.794)	0.001	0.560 (0.372–0.844)	0.006
**Marital status**				
Married	1.0 (referent)			
Other	1.107 (0.932–1.315)	0.247		
**Diagnosis Year (continuous variable)**	0.970 (0.954–0.986)	**<0.001**	0.971 (0.952–0.991)	**0.004**
**Tumor location**		0.738		
Pancreatic Head	1.0 (referent)			
Other	0.971 (0.817–1.154)			
**T stage**		**<0.001**		**<0.001**
T1	1.0 (referent)		1.0 (referent)	
T2	1.639 (1.102–2.437)	0.015	1.601 (0.961–2.668)	0.071
T3	3.418 (2.393–4.882)	<0.001	2.352 (1.457–3.796)	**<0.001**
T4	5.630 (3.426–9.252)	<0.001	5.492 (2.921–10.325)	**<0.001**
**N stage**		**<0.001**		**<0.001**
N0	1.0 (referent)		1.0 (referent)	
N1	2.487 (1.887–3.279)		2.192 (1.732–2.774)	
**M stage**		**<0.001**		**0.005**
M0	1.0 (referent)		1.0 (referent)	
M1	2.589 (1.926–3.481)		2.017 (1.239–3.282)	
**Histological Grade**		**<0.001**		**<0.001**
Well differentiated; Grade I	1.0 (referent)		1.0 (referent)	
Moderately differentiated; Grade II	1.794 (1.410–2.282)	**<0.001**	1.609 (1.242–2.083)	**<0.001**
Poorly differentiated; Grade III	2.659 (2.031–3.480)	**<0.001**	2.077 (1.548–2.787)	**<0.001**
Undifferentiated; Grade III	4.057 (1.768–9.308)	**<0.001**	2.460 (0.563–10.742)	0.231
**Surgery Type**		0.860		
Pancreatoduodenectomy	1.0 (referent)			
Total pancreatectomy	1.000 (0.777–1.288)	0.999		
Partial or Local pancreatectomy	0.940 (0.751–1.176)	0.589		
**Radiation therapy**		**0.014**		0.056
Not Received	1.0 (referent)		1.0 (referent)	
Received	1.236 (1.043–1.464)		0.816 (0.662–1.005)	
**Lymph node count (continuous variable)**	0.980 (0.970–0.990)	**<0.001**	0.966 (0.952–0.979)	**<0.001**
**No. positive LN**	1.073 (1.053–1.092)	**<0.001**	1.040 (1.004–1.078)	0.031

### The Lymph Node Count Cut-off Value Determination

**Figure 1:**
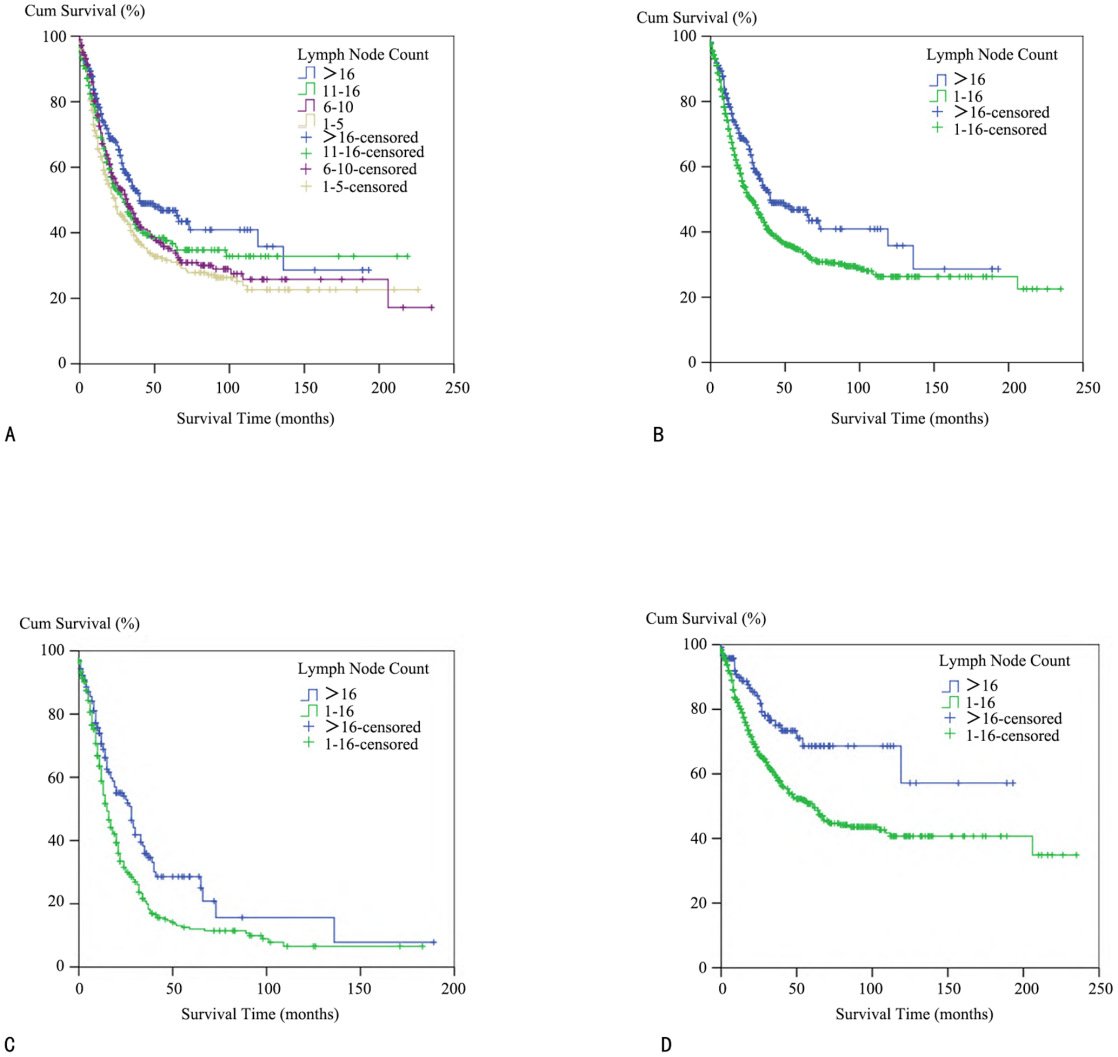
Kaplan–Meier survival analysis for invasive IPMN patients with different lymph node counts. (A) A cancer-specific survival (CSS) curve for patients with lymph node count of 1–5, 6–10, 11–16 and over 16. (B) A cancer-specific survival (CSS) curve for patients with lymph node counts of 1–16 and over 16. (C) A cancer-specific survival (CSS) curve for N1 patients with lymph node counts of 1–16 and over 16. (D) A cancer-specific survival (CSS) curve for N0 patients with lymph node counts of 1–16 and over 16.

We utilized the Kaplan–Meier method to study the CSS of the patients by four lymph node count subgroups to decipher the most informative lymph node count cut-off (Figure 1A). Log-rank tests were implemented pairwise over each subgroup to test potential significant differences. No significant differences were observed between subgroup 1, 2, and 3, but there was a significant difference between subgroup 4 and subgroups 1/2/3 (subgroup 1–4, p<0.001; subgroup 2–4, p = 0.036; subgroup 3–4, p = 0.033). This suggests that dissection of more than 16 lymph nodes can significantly improve CSS, therefore extending the IPMN survival time. Following this finding, we combined subgroups 1/2/3 together and then assessed the effects from different lymph node dissection numbers (1–16 vs. above 16). The data showed that in the group where over 16 nodes were dissected, the median survival time was 40 months, while in the group where 1 to 16 nodes were dissected, the median survival time was only 28 months. This difference was significantly different, with a P value of 0.002 (Figure 1B). To validate the correlation between the lymph node count and survival, we conducted a multivariate Cox analysis using the number of dissected lymph nodes as a categorical variable. First, we dichotomously defined the number of dissected lymph nodes (1–16 vs.>16) as previously discussed. Then, we observed that HR was 0.662 in the>16 group, suggesting an HR reduction when more than 16 lymph nodes were dissected (95% CI: 0.514–0.853, p = 0.001). For the N0 patients, the HR was 0.568 when more than 16 nodes were dissected (95% CI: 0.348–0.928, p = 0.024). Additionally, for the N1 patients, the HR was 0.728 when more than 16 nodes were dissected (95% CI: 0.539–0.982, p = 0.038) (Table S2).

### Survival Time in Different T, N Stages and Histological Grade

Patient survival time is strongly influenced by various factors such as T, N stages and histological grades. To further investigate the survival in different T, N stages and histological grades, separate subgroup analyses were performed.

Among the N1 population, the median survival time was 28 months in the>16 node group, and 15 months in the 1–16 node group (P<0.001, Figure 1C). Among the N0 population, more than half of the>16 node group patients still were alive at the time of analysis; therefore, the median survival time was not available. The 1–16 node group, however, had a median survival time of 61 months. The P value between these groups was less than 0.001, which represented a significant difference (Figure 1D).

Among the T1 population, more than half of the>16 node group patients still were alive at the time of analysis; therefore, the median survival time was not available. Meanwhile, the survival is 112 months in the 1–16 node group (P = 0.122, Figure S1, Panel A). Among the T2 population, more than half of the>16 node group patients still were alive at the time of analysis; therefore, the median survival time was not available. The 1–16 node group, however, had a median survival time of 64 months. The P value between these groups was 0.031, which represented a significant difference (Figure S1, Panel B). Among the T3 population, the median survival time was 29 months in>16 node group, and 20 months in 1–16 node group. The P value between these groups was 0.071 (Figure S1, Panel C).

Similarly, the median survival is 119 months and 61 months in>16 node group and 1–16 node group for Grade I patients, respectively (P value  = 0.489, Figure S1, Panel D). The median survival is 35 months and 22 months in>16 node group and 1–16 node group for Grade II patients, respectively (P value  = 0.134, Figure S1, Panel E). The median survival time is 26 months and 13 months in>16 node group and 1–16 node group for Grade III patients, respectively (P value  = 0.007, Figure S1, Panel F).

### Stage Migration Effect Detection

To evaluate the stage migration effect in this cohort, we first explored the number and the proportion of N stage patients in each of the four lymph node intervals (Table 3). There were trends towards low proportion of patients with N0 and higher proportion of patients with N1 when lymph node count increased. The chi-square tests showed that this trend was statistically significant when including patients of both N0 and N1 stage (P value <0.001). To explore whether patients with different lymph node count intervals had similar disease severity, the distribution of T stage and histological grade among different lymph node count intervals were analyzed (Table S3 and Table S4). The chi-square tests showed that no statistically significantly difference were observed in T stage distribution (T1, T2 and T3) (P value  = 0.102). The chi-square tests also showed that histological grade distribution (Grade I, II, III) was not significantly different among different lymph node count intervals (P value  = 0.201). We did not include T4 stage or Grade IV patients in either of these chi-square tests because of the relative small number of patients in these two categories. Thus, we concluded that patients of different lymph node intervals did not show significant difference in disease severity, as illustrated by T stage and histological grade distributions. Stage migration occurred in this cohort. Patients with more lymph node dissected had greater chance to be accurately staged for the lymph node status.

**Table 3:** The Number and Percentage of N Stage for Different Total Nodal Count Intervals for Invasive IPMN Patients: Surveillance, Epidemiology, and End Results 1992 to 2011.

No. Dissected Nodes	N stage		
	N0	N1	NX*
1–5	184 (0.66)	93 (0.33)	3 (0.01)
6–10	156 (0.55)	122 (0.43)	4 (0.01)
11–16	128 (0.50)	127 (0.49)	2 (0.01)
>16	120 (0.46)	141 (0.54)	0 (0.00)

*NX: N stage was undefined in the Surveillance, Epidemiology, and End Results database.

### Testing the Correlation between the Number of Dissected Lymph Nodes and Survival Time by Propensity Score Matching (PSM)

**Figure 2:**
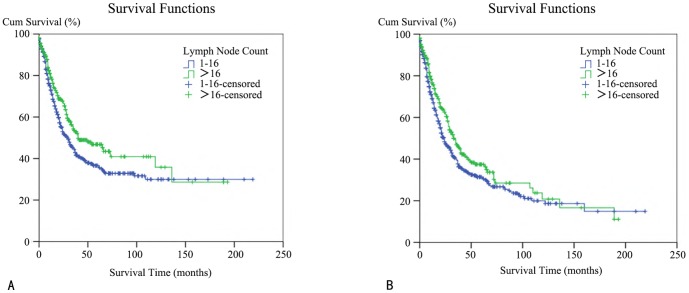
A Kaplan–Meier survival analysis for invasive IPMN patients after propensity score matching with different lymph node counts. (A) A cancer-specific survival (CSS) curve for patients with lymph node counts of 1–16 and over 16 is shown. (B) An overall survival (OS) curve for patients with lymph node counts of 1–16 and over 16 is shown.

We utilized PSM to balance potential confounding factors, including age, gender, race, marital status, diagnosis year, tumor location, T stage, M stage, histological grade, and surgery and radiation types. The N stage was not included as a covariate because it was influenced by, and not completely independent of the lymph node count. During the matching process, patients in the 1–16 lymph node group were matched with those in the>16 lymph node group with a ratio of 2∶1. The baseline characteristics in two groups before and after match were demonstrated in Table S5. Then, we performed a Kaplan-Meier analysis on the patients after the match to address the CSS and OS rates in either the>16 group or the 1–16 group. Thus, we arrived at a conclusion that was in concordance with our previous assessment. We first focused on the CSS assessment. The>16 group had a median survival time of 40 months, whereas the 1–16 group had a median survival time of just 30 months; therefore, the absolute elevation was 10 months. In this log-rank test, the P value was 0.011. Additionally, the>16 group had a 47% five-year survival rate, whereas the 1–16 group only had a 36% five-year survival rate; therefore, the absolute elevation was 11% (Figure 2A). Next, we evaluated the OS. The>16 group had a median survival time of 33 months whereas the 1–16 group had a median survival time of just 24 months; therefore, the absolute elevation was 9 months. Following a log-rank test for this comparison, the P value was 0.026. Additionally, the>16 group had a 37% five-year survival rate, whereas in the 1–16 group only had a 31% five-year survival rate; therefore, the absolute elevation was 6% (Figure 2B).

## Discussion

IPMNs can be grossly sub-classified into benign and malignant tumors in accordance with the World Health Organization histological criteria [1]. Malignant IPMNs can be further divided into carcinoma in situ and invasive cancer. Invasive IPMNs usually carry worse prognosis than non-invasive IPMNs. The lymph node status is a strong predictor for poor survival of patients with invasive IPMNs, which was observed and validated by independent cohorts across different medical facilities over the past years. C Azar et al reported no pancreas-related mortality in 15 non-invasive IPMN patients, whereas four out of nine invasive IPMN patients died within 6 months, postoperatively [29]. A Johns Hopkins series consisted of a total of 136 IPMN patients. Fifty-two patients (38%) in this series were diagnosed with an invasive IPMN. The five-year overall survival rate was 77% and 43% for the non-invasive and invasive IPMN patients, respectively. For those with invasive IPMNs, the five-year overall survival rate was 85% and 0% for lymph node negative and positive patients, respectively [18], [30]. Joint research by Massachusetts General Hospital and the University of Verona Pancreatic Unit found that the disease-specific survival rate was 100% and 60% for non-invasive and invasive main-duct IPMN patients, respectively. Though not statistically significant, the five-year actuarial survival rate was found to be lower for lymph node positive patients [9], [31]. A subsequent analysis based on 104 patients with invasive IPMN revealed that the lymph node ratio could be a predictor for survival. A significant correlation was observed between an increased lymph node ratio and a decreased five-year disease specific survival rate [23]. A study based on a Mayo clinic case series exhibited a similar trend towards worse survival for invasive IPMN patients than their non-invasive counterparts (the five-year overall survival rates were 94% versus 31%, respectively). Invasive IPMNs were also associated with a higher incidence of disease recurrence (58%) compared with a non-invasive group (10%). A subgroup analysis, including only invasive IPMNs, showed that lymph node involvement was an adverse prognostic factor for survival [17], [19]. Four French medical centers initiated studies involving 73 malignant IPMN patients. The five-year survival rate was 88% and 36% for carcinoma in situ and invasive IPMN patients, respectively. Of 51 invasive IPMN patients, lymph node invasion was the only independent factor that predicted worse survival in that cohort [32]. A similar result was also shown in a Memorial Sloan-Kettering Cancer Center study, in which survival was significantly worse in invasive IPMN patients and lymph node invasion further worsened the prognosis for invasive IPMN patients [21]. The results from present SEER database analysis are consistent with previous reports. The five-year CSS for all of the invasive IPMN patients was 37%. Additionally, the five-year CSS for N0 and N1 invasive IPMN patients was 54% and 16%, respectively.

Since lymph node metastasis predisposes patients to a worse prognosis, one would speculate whether increasing the total number of lymph node harvested during surgery would be of any benefit for survival. On one hand, lymph node dissections help remove suspicious lymph nodes that may contain micrometastasis, therefore, preventing disease recurrence via lymphatic spread. On the other hand, patients with extended lymph node dissections are prone to postoperative complications, which can hamper long-term survival. Although lymph node metastasis is proved to be an adverse prognostic factor for various gastrointestinal malignancies, the therapeutic effects of lymph node dissections are quite different across different tumor types. Increased lymph node dissections improve the survival for colon and rectal cancer patients [33]–[35]. For gastric cancer, extended lymph node dissections (D2 dissection) are recommended only in high-volume medical facilities by experienced surgeons [36]. As for pancreatic adenocarcinoma, extended lymphadenectomy should be avoided because no survival benefits have been observed so far [37].

Approximately two-thirds of patients did not live for over five years in this study, even after an initial surgery for invasive IPMNs. One of the reasons is disease recurrence, which hampered the patients' long-term survival. Distant recurrences are more common than remnant pancreas recurrences; the liver, peritoneum and lymph nodes are at high risks of recurrence [17], [18], [21]. It is not known whether increasing lymph node dissections would reduce the distant metastasis and prolong survival times. The relative low incidence of this disease makes it difficult to initiate randomized controlled trials for this issue. Retrospective databases may provide a sufficient number of patients to reach statistical power for determining if extended lymph node dissection really benefits survival. However, because patient survival is strongly affected by other prognostic factors as well, such as TNM staging and histology grades, one should try best to minimize the influence of these confounding factors. The present analysis utilized both Kaplan-Meier survival and Cox-regression multivariate survival analyses to evaluate the impact of lymph node dissections on survival. Sixteen lymph node dissections were proposed to be the cut-off value. The patients whose lymph node counts were over this cut-off value showed a significant survival benefit. A subgroup analysis of either lymph node positive or lymph node negative patients showed benefits from lymph node counts over this cut-off value. Additional, T2 and Grade III patients also significantly benefit from extended lymph node dissection. To validate this finding and minimizing the influence of any confounding variables, we further conducted a propensity-score matching analysis after which all potential confounding variables were balanced between the patients with a>16 lymph node count and those with no more than a 16 lymph node count. An absolute median survival elevation of 10 and 9 months were obtained regarding the cause-specific and overall survival, respectively. Additionally, an absolute elevation of 11% and 6% were obtained regarding the 5-year cause-specific and 5-year overall survival, respectively.

We were also interested in the minimal lymph node counts required for the accurate staging of invasive IPMNs. Thus, we investigated the stage migration effect, which referred to the situation where an increased lymph node count would increase the chance of detecting a metastatic lymph node [38]. In order to accurately stage the disease, a minimally required lymph node count was proposed for some gastrointestinal malignancies, including gastric and colon cancers [39], [40]. We analyzed the N stage distribution across four different patient subgroups with increasing lymph node counts. A statistically significant trend was observed that increased lymph node count correlates with larger proportion of N1 patients. This phenomenon could be interpreted by either of the following two reasons. One possibility is that patients with fewer lymph nodes dissected were understaged; therefore more lymph node dissection would help find positive lymph nodes. The other possibility is that patients with more lymph nodes dissected were more likely to have more severe diseases and harbor lymph node metastasis. Although we could not definitely rule out either of the two possibilities, we tried to assess disease severity by analyzing T stages and histological stage distributions among different lymph node intervals. Both of them were evenly distributed in each lymph node intervals. Therefore, patients with more lymph node count had similar disease severity with those with less lymph node count. The apparent increased proportion of N1 patients for more lymph node dissection should mainly explained by the stage migration effect.

We acknowledge there are certain inherent limitations of the present analysis. First, it is critical to address the issue of whether extended lymph node dissections would prolong disease-free survival and reduce the disease recurrence incidence. Other variables, such as the IPMN type (main-duct, branch-duct, or mixed-type), chemotherapy status, and margin status, may potentially influence survival rate. Nonetheless, these variables are not available from the SEER database, alone. Second, the case list was derived based on the ICD-O-3 codes for invasive IPMNs. One should recognize the variability of the pathologic diagnosis across different institutions and time periods, which may lead to misdiagnosed and undiagnosed invasive IPMN patients. Third, the selection bias cannot be completely avoided even when we used propensity score matching to balance baseline variables between patients with fewer lymph node dissections and those with relatively more dissections.

In conclusion, more lymph nodes should be harvested during invasive IPMN surgery. First, lymph node dissections with over 16 lymph nodes are more likely to benefit survival. Second, it could help avoid understaging of lymph node metastatic status.

## Supporting Information

Figure S1 **Kaplan–Meier survival analysis for invasive IPMN patients with different T stages and histological grades.** (A) A cancer- specific survival (CSS) curve for T1 patients with lymph node count of 1-16 and over 16. (B) A cancer-specific survival (CSS) curve for T2 patients with lymph node count of 1-16 and over 16. (C) A cancer-specific survival (CSS) curve for T3 patients with lymph node count of 1-16 and over 16. (D) A cancer-specific survival (CSS) curve for histological Grade I patients with lymph node count of 1-16 and over 16. (E) A cancer-specific survival (CSS) curve for histological Grade II patients with lymph node count of 1–16 and over 16. (F) A cancer- specific survival (CSS) curve for histological Grade III patients with lymph node count of 1–16 and over 16.
(TIF)

Table S1 **Variance Inflation Factors (VIF) Calculation For All Variables in Invasive IPMN Patients: Surveillance, Epidemiology, and End Results 1992 to 2011.**
(DOCX)

Table S2 **Multivariate Cox Proportional Hazard Regression Analyses for Cancer-Specific Survival for All, N0 and N1 Invasive IPMN Patients: Surveillance, Epidemiology, and End Results 1992 to 2011.**
(DOCX)

Table S3 **The Number and Percentage of T Stage for Different Total Nodal Count Intervals for Invasive IPMN Patients: Surveillance, Epidemiology, and End Results 1992 to 2011.** *TX: T stage was undefined in the Surveillance, Epidemiology, and End Results database.
(DOCX)

Table S4 **The Number and Percentage of Histological Grade for Different Total Nodal Count Intervals for Invasive IPMN Patients: Surveillance, Epidemiology, and End Results 1992 to 2011.**
(DOCX)

Table S5 **Patient, Tumor, and Treatment Characteristics in the Invasive IPMN Patients: Surveillance, Epidemiology, and End Results, 1992 to 2011.** * P value was calculated by Chi-square tests for the categorical variables. Unknown value was not included into the Chi-square tests. Student's t-tests were used for continuous variables † For histological grade, only Grade I-Grade III were included for chi-square tests because of the relative fewer cases in G4 groups.
(DOCX)
