# Supplementary figures and images for: An Increased Total Resected Lymph Node Count Benefits Survival following Pancreas Invasive Intraductal Papillary Mucinous Neoplasms Resection: An Analysis Using the Surveillance, Epidemiology, and End Result Registry Database
Source: PLoS One. 2014 Sep 29;9(9):e107962. doi: 10.1371/journal.pone.0107962 (PMC4179272; doi:10.1371/journal.pone.0107962)

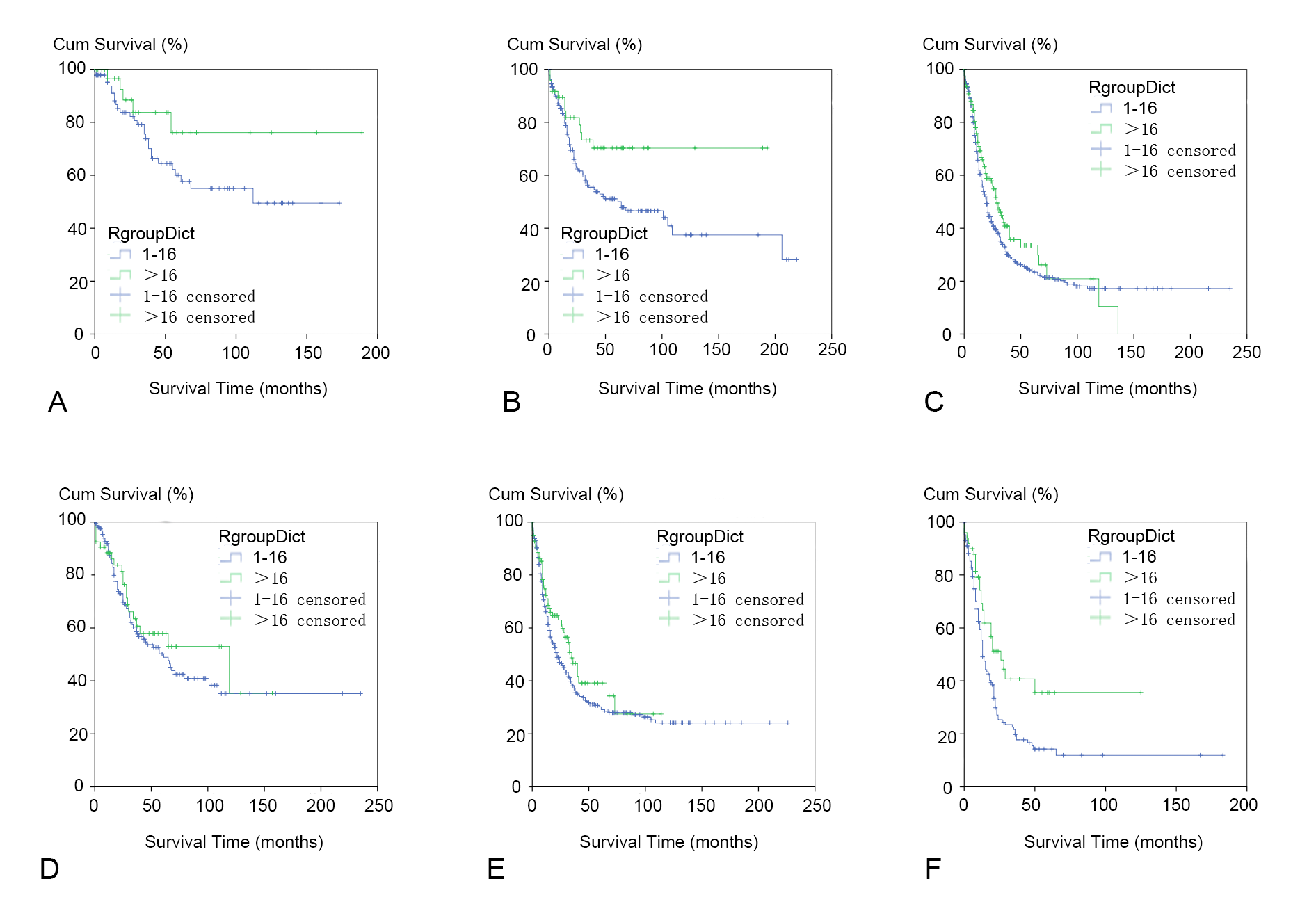

Supplement: Figure S1 — Kaplan–Meier survival analysis for invasive IPMN patients with different T stages and histological grades. (A) A cancer- specific survival (CSS) curve for T1 patients with lymph node count of 1-16 and over 16. (B) A cancer-specific survival (CSS) curve for T2 patients with lymph node count of 1-16 and over 16. (C) A cancer-specific survival (CSS) curve for T3 patients with lymph node count of 1-16 and over 16. (D) A cancer-specific survival (CSS) curve for histological Grade I patients with lymph node count of 1-16 and over 16. (E) A cancer-specific survival (CSS) curve for histological Grade II patients with lymph node count of 1–16 and over 16. (F) A cancer- specific survival (CSS) curve for histological Grade III patients with lymph node count of 1–16 and over 16. (TIF) [file pone.0107962.s001.tif]
