# Supplementary material for: An Increased Total Resected Lymph Node Count Benefits Survival following Pancreas Invasive Intraductal Papillary Mucinous Neoplasms Resection: An Analysis Using the Surveillance, Epidemiology, and End Result Registry Database
Source: PLoS One. 2014 Sep 29;9(9):e107962. doi: 10.1371/journal.pone.0107962 (PMC4179272; doi:10.1371/journal.pone.0107962)
Supplement: Table S1 — Variance Inflation Factors (VIF) Calculation For All Variables in Invasive IPMN Patients: Surveillance, Epidemiology, and End Results 1992 to 2011. (DOCX) [file pone.0107962.s002.docx]

Table S1. Variance Inflation Factors (VIF) Calculation For All Variables in Invasive IPMN Patients: Surveillance, Epidemiology, and End Results 1992 to 2011.

| Variables | Before Exclusion of AJCC stage | | After Exclusion of AJCC stage | |
| --- | --- | --- | --- | --- |
|  | Tolerance | VIF | Tolerance | VIF |
| Age, y (continuous variable) | 0.947 | 1.056 | 0.949 | 1.054 |
| Sex | 0.918 | 1.090 | 0.918 | 1.089 |
| Race | 0.984 | 1.016 | 0.985 | 1.015 |
| Marital Status | 0.926 | 1.080 | 0.928 | 1.078 |
| Diagnosis Year (Continuous Variable) | 0.907 | 1.103 | 0.907 | 1.102 |
| Tumor Location | 0.693 | 1.444 | 0.700 | 1.429 |
| T Stage | 0.316 | 3.164 | 0.866 | 1.154 |
| N Stage | 0.536 | 1.867 | 0.625 | 1.600 |
| M Stage | 0.309 | 3.235 | 0.951 | 1.051 |
| AJCC Stage | 0.158 | **6.317** |  |  |
| Histological Grade | 0.911 | 1.098 | 0.911 | 1.098 |
| Surgery Type | 0.716 | 1.397 | 0.719 | 1.392 |
| Radiation Therapy | 0.872 | 1.147 | 0.875 | 1.143 |
| Lymph Node Count (Continuous Variable) | 0.856 | 1.168 | 0.858 | 1.165 |
| No. Positive LN | 0.643 | 1.556 | 0.646 | 1.547 |
