# Supplementary material for: An Increased Total Resected Lymph Node Count Benefits Survival following Pancreas Invasive Intraductal Papillary Mucinous Neoplasms Resection: An Analysis Using the Surveillance, Epidemiology, and End Result Registry Database
Source: PLoS One. 2014 Sep 29;9(9):e107962. doi: 10.1371/journal.pone.0107962 (PMC4179272; doi:10.1371/journal.pone.0107962)
Supplement: Table S2 — Multivariate Cox Proportional Hazard Regression Analyses for Cancer-Specific Survival for All, N0 and N1 Invasive IPMN Patients: Surveillance, Epidemiology, and End Results 1992 to 2011. (DOCX) [file pone.0107962.s003.docx]

Table S2. Multivariate Cox Proportional Hazard Regression Analyses for Cancer-Specific Survival for All, N0 and N1 Invasive IPMN Patients: Surveillance, Epidemiology, and End Results 1992 to 2011.

| Column | All patients | | N0 patients | | N1 patients | |
| --- | --- | --- | --- | --- | --- | --- |
|  | HR (95% CI) | P Value | HR (95% CI) | P Value | HR (95% CI) | P Value |
| Age (Continuous Variable) | 1.016 (1.007-1.025) | **<0.001** | 1.023 (1.008-1.038) | **0.002** | 1.010 (0.999-1.022) | 0.074 |
| Race |  | **0.019** |  | 0.052 |  | 0.369 |
| White (Reference) | 1.0 (referent) |  | 1.0 (referent) |  | 1.0 (referent) |  |
| Black | 0.938 (0.642-1.370) | 0.740 | 1.091 (0.582-2.044) | 0.787 | 0.922 (0.568-1.495) | 0.741 |
| Other (American Indian/Ak Native, Asian/Pacific Islander) | 0.548 (0.361-0.833) | **0.005** | 0.385 (0.176-0.840) | **0.016** | 0.704 (0.430-1.152) | 0.163 |
| Year of Diagnosis (Continuous Variable) | 0.966 (0.947-0.986) | **0.001** | 0.950 (0.920-0.982) | **0.002** | 0.975 (0.950-1.001) | 0.055 |
| T Stage |  | **0.007** |  | **<0.001** |  | 0.118 |
| T1 (Reference) | 1.0 (referent) |  | 1.0 (referent) |  | 1.0 (referent) |  |
| T2 | 1.599 (0.948-2.699) | 0.079 | 1.952 (1.025-3.716) | 0.042 | 0.974 (0.404-2.347) | 0.952 |
| T3 | 2.022 (1.132-3.613) | 0.017 | 2.772 (1.522-5.047) | 0.001 | 1.258 (0.552-2.871) | 0.585 |
| T4 | 6.954 (2.233-21.658) | 0.001 | 10.997 (4.716-25.645) | <0.001 | 2.225 (0.808-6.129) | 0.122 |
| Lymph Node Involvement |  | **<0.001** |  |  |  |  |
| N0 | 1.0 (referent) |  | N/A |  | N/A |  |
| N1 | 2.056 (1.586-2.664) |  | N/A |  | N/A |  |
| Metastasis |  | **0.029** |  | 0.088 |  | **0.036** |
| M0 (Reference) | 1.0 (referent) |  | 1.0 (referent) |  | 1.0 (referent) |  |
| M1 | 2.230 (1.087-4.576) |  | 2.013 (0.900-4.500) |  | 1.936 (1.043-3.593) |  |
| Pathologic Grade |  | **<0.001** |  | **<0.001** |  | **0.011** |
| Grade I (Reference) | 1.0 (referent) |  | 1.0 (referent) |  | 1.0 (referent) |  |
| Grade II | 1.594 (1.230-2.066) | <0.001 | 1.386 (0.939-2.044) | 0.100 | 1.800 (1.256-2.579) | 0.001 |
| Grade III | 2.057 (1.532-2.761) | <0.001 | 2.681 (1.683-4.270) | <0.001 | 1.794 (1.216-2.647) | 0.003 |
| Grade IV | 2.707 (0.616-11.893) | 0.187 | 9.440 (1.193-74.697) | 0.033 | 1.317 (0.164-10.570) | 0.796 |
| Radiation Therapy |  | **0.031** |  | 0.458 |  | **<0.001** |
| No (Reference) | 1.0 (referent) |  | 1.0 (referent) |  | 1.0 (referent) |  |
| Yes | 0.795 (0.645-0.979) |  | 1.143 (0.803-1.629) |  | 0.619 (0.478-0.801) |  |
| No. Positive LN | 1.026 (0.991-1.063) | 0.146 | 0.800 (0.585-1.093) | 0.161 | 1.029 (0.992-1.068) | 0.129 |
| Number of LNs Examined |  | **0.001** |  | **0.024** |  | **0.038** |
| 1-16 | 1.0 (referent) |  | 1.0 (referent) |  | 1.0 (referent) |  |
| > 16 | 0.662 (0.514-0.853) |  | 0.568 (0.348-0.928) |  | 0.728 (0.539-0.982) |  |
