# Supplementary material for: An Increased Total Resected Lymph Node Count Benefits Survival following Pancreas Invasive Intraductal Papillary Mucinous Neoplasms Resection: An Analysis Using the Surveillance, Epidemiology, and End Result Registry Database
Source: PLoS One. 2014 Sep 29;9(9):e107962. doi: 10.1371/journal.pone.0107962 (PMC4179272; doi:10.1371/journal.pone.0107962)
Supplement: Table S3 — The Number and Percentage of T Stage for Different Total Nodal Count Intervals for Invasive IPMN Patients: Surveillance, Epidemiology, and End Results 1992 to 2011. *TX: T stage was undefined in the Surveillance, Epidemiology, and End Results database. (DOCX) [file pone.0107962.s004.docx]

Table S3. The Number and Percentage of T Stage for Different Total Nodal Count Intervals for Invasive IPMN Patients: Surveillance, Epidemiology, and End Results 1992 to 2011.

| No. Dissected Nodes | T stage | | | | |
| --- | --- | --- | --- | --- | --- |
|  | T1 | T2 | T3 | T4 | TX* |
| 1-5 | 36 (0.13) | 67 (0.24) | 136 (0.49) | 16 (0.06) | 25 (0.09) |
| 6-10 | 29 (0.10) | 59 (0.21) | 168 (0.60) | 4 (0.01) | 22 (0.08) |
| 11-16 | 28 (0.11) | 58 (0.23) | 151 (0.59) | 8 (0.03) | 12 (0.05) |
| >16 | 36 (0.14) | 48 (0.18) | 154 (0.59) | 12 (0.05) | 11 (0.04) |

*TX: T stage was undefined in the Surveillance, Epidemiology, and End Results database
