# Supplementary material for: An Increased Total Resected Lymph Node Count Benefits Survival following Pancreas Invasive Intraductal Papillary Mucinous Neoplasms Resection: An Analysis Using the Surveillance, Epidemiology, and End Result Registry Database
Source: PLoS One. 2014 Sep 29;9(9):e107962. doi: 10.1371/journal.pone.0107962 (PMC4179272; doi:10.1371/journal.pone.0107962)
Supplement: Table S4 — The Number and Percentage of Histological Grade for Different Total Nodal Count Intervals for Invasive IPMN Patients: Surveillance, Epidemiology, and End Results 1992 to 2011. (DOCX) [file pone.0107962.s005.docx]

Table S4. The Number and Percentage of Histological Grade for Different Total Nodal Count Intervals for Invasive IPMN Patients: Surveillance, Epidemiology, and End Results 1992 to 2011.

| No. Dissected Nodes | Histological Grade | | | | |
| --- | --- | --- | --- | --- | --- |
|  | Grade1 | Grade II | Grade III | Grade IV | Unknown |
| 1-5 | 54 (0.19) | 117 (0.42) | 38 (0.14) | 4 (0.01) | 67 (0.24) |
| 6-10 | 64 (0.23) | 108 (0.38) | 52 (0.18) | 3 (0.01) | 55 (0.20) |
| 11-16 | 42 (0.16) | 98 (0.38) | 56 (0.22) | 1 (0.00) | 60 (0.23) |
| >16 | 53 (0.20) | 98 (0.38) | 50 (0.19) | 3 (0.01) | 57 (0.22) |
