# Supplementary material for: An Increased Total Resected Lymph Node Count Benefits Survival following Pancreas Invasive Intraductal Papillary Mucinous Neoplasms Resection: An Analysis Using the Surveillance, Epidemiology, and End Result Registry Database
Source: PLoS One. 2014 Sep 29;9(9):e107962. doi: 10.1371/journal.pone.0107962 (PMC4179272; doi:10.1371/journal.pone.0107962)
Supplement: Table S5 — Patient, Tumor, and Treatment Characteristics in the Invasive IPMN Patients: Surveillance, Epidemiology, and End Results, 1992 to 2011. * P value was calculated by Chi-square tests for the categorical variables. Unknown value was not included into the Chi-square tests. Student's t-tests were used for continuous variables † For histological grade, only Grade I-Grade III were included for chi-square tests because of the relative fewer cases in G4 groups. (DOCX) [file pone.0107962.s006.docx]

Table S5. Patient, Tumor, and Treatment Characteristics in the Invasive IPMN Patients: Surveillance, Epidemiology, and End Results, 1992 to 2011

| Variable | Before Propensity Score Matching | | | After Propensity Score Matching | | |
| --- | --- | --- | --- | --- | --- | --- |
|  | Number of Patients / Mean (Standard Deviation) | | P Value* | Number of Patients / Mean (Standard Deviation) | | P Value |
|  | 1-16 group | > 16 group |  | 1-16 group | > 16 group |  |
| **Age** | 65.4 (12.5) | 64.5 (10.9) | **0.032** | 65.3 (12.6) | 64.5 (10.9) | **0.046** |
| **Sex** |  |  | 0.818 |  |  | 0.839 |
| Women | 373 | 121 |  | 246 | 121 |  |
| Men | 446 | 140 |  | 276 | 140 |  |
| **Race** |  |  | 0.298 |  |  | 0.215 |
| White | 690 | 217 |  | 441 | 217 |  |
| Black | 54 | 24 |  | 32 | 24 |  |
| Other (American Indian/AK Native, Asian/Pacific Islander) | 72 | 19 |  | 48 | 19 |  |
| Unknown | 3 | 1 |  | 1 | 1 |  |
| **Marital status** |  |  | 0.967 |  |  | 1.000 |
| Married | 271 | 86 |  | 172 | 86 |  |
| Other | 548 | 175 |  | 350 | 175 |  |
| **Diagnosis Years Intervals** | 2003.6 (5.1) | 2005.8 (4.6) | **0.009** | 2005.5 (4.3) | 2005.8 (4.6) | 0.294 |
| **Tumor location** |  |  | **0.023** |  |  | 0.697 |
| Pancreatic Head | 524 | 187 |  | 367 | 187 |  |
| Other | 295 | 74 |  | 155 | 74 |  |
| **T stage** |  |  | 0.33 |  |  | 0.252 |
| T1 | 93 | 36 |  | 70 | 36 |  |
| T2 | 184 | 48 |  | 128 | 48 |  |
| T3 | 455 | 154 |  | 278 | 154 |  |
| T4 | 28 | 12 |  | 21 | 12 |  |
| Unknown | 59 | 11 |  | 25 | 11 |  |
| **M stage** |  |  | 0.073 |  |  | 0.110 |
| M0 | 758 | 247 |  | 484 | 247 |  |
| M1 | 57 | 10 |  | 35 | 10 |  |
| Unknown | 4 | 4 |  | 3 | 4 |  |
| **Histological Grade** † |  |  | 0.806 |  |  | 0.681 |
| Well differentiated; Grade I | 160 | 53 |  | 100 | 53 |  |
| Moderately differentiated; Grade II | 323 | 98 |  | 211 | 98 |  |
| Poorly differentiated; Grade III | 146 | 50 |  | 91 | 50 |  |
| Undifferentiated; Grade IV | 8 | 3 |  | 4 | 3 |  |
| Unknown | 182 | 57 |  | 116 | 57 |  |
| **Surgery Type** |  |  | 0.059 |  |  | 0.453 |
| Pancreatoduodenectomy | 559 | 175 |  | 362 | 175 |  |
| Total pancreatectomy | 94 | 43 | S | 69 | 43 |  |
| Partial or Local pancreatectomy | 144 | 36 |  | 78 | 36 |  |
| Other | 22 | 7 |  | 13 | 7 |  |
| **Radiation therapy** |  |  | 0.885 |  |  | 0.257 |
| Received | 258 | 85 |  | 148 | 85 |  |
| Not Received | 543 | 175 |  | 367 | 175 |  |
| Unknown | 18 | 1 |  | 7 | 1 |  |

* P value was calculated by Chi-square tests for the categorical variables. Unknown value was not included into the Chi-square tests.Student's t-tests were used for continuous variables

† For histological grade, only Grade I-Grade III were included for chi-square tests because of the relative fewer cases in Grade IV groups
